# Retrieval of HCV patients lost to follow-up as a strategy for Hepatitis C Microelimination: results of a Brazilian multicentre study

**DOI:** 10.1186/s12879-023-08169-0

**Published:** 2023-07-13

**Authors:** Maria Lucia Gomes Ferraz, Antonio Ricardo Cardia Ferraz de Andrade, Gustavo Henrique Santos Pereira, Liana Codes, Paulo Lisboa Bittencourt

**Affiliations:** 1 Federal University of São Paulo, Rua Loefgren 1570, São Paulo, CEP 04040-002 SP Brazil; 2 Federal University of Bahia, Salvador, Brazil; 3 Federal Hospital of Bonsucesso, Rio de Janeiro, Brazil; 4 School of Medicine (IDOMED), Estácio de Sá University, Rio de Janeiro, Brazil; 5 Bahiana School of Medicine and Public Health, Bahia, Brazil; 6 Portuguese Hospital, Bahia, Brazil

**Keywords:** Hepatitis C, DAA treatment, Non-SVR, Micro-elimination

## Abstract

**Background:**

Several HCV patients in Brazil were lost to follow-up (LTFU) in the last two decades before achievement of sustained virological response (SVR). Strategies to recall those diagnosed but untreated patients have been used elsewhere with different success rates.

**Aim:**

To identify and retrieve LTFU patients in order to offer them the treatment with the current highly effective direct acting antiviral agents (DAAs).

**Methods:**

Registries ofall HCV patients from three large reference centers in Brazil were retrospectively reviewed to identify those with no registry of SVR. Reasons for non-achievement of SVR were elicited in HCV-RNA + patients. All patients who were not treated or cured were contacted to offer the therapy with DAAs.

**Results:**

10,289 HCV patients (50% males, mean age 52 ± 11 years) were identified. Only 4,293 (41.7%) had been successfully treated previously. From the remaining 5,996 most were LTFU (59%), were not treated for other reasons (14.7%) or were non-responders (26.3%). After revision of the charts 3,559 were considered eligible to be retrieved. The callback success of phone calls was 18%, 13% to cellphone messages (SMS or WhatsApp) and 7% to regular mail. Five-hundred sixty patients had been already treatedor were on treatment and 234 were reported to be dead or transplanted. Finally, 201 had made an appointment and initiated antiviral treatment.

**Conclusion:**

Even considering the low callback rate, retrieval of LTFU patients was shown to be an important strategy forhepatitis C micro-elimination in Brazil.

## Introduction

Hepatitis C virus (HCV) infection affects almost 58 million of people globally [1] and is still one of the most common causes of cirrhosis and hepatocellular carcinoma (HCC) in the Western world, responsible for approximately 290,000 deaths in 2019, mostly in those HCV + subjects not submitted to treatment {2]. Fortunately, with the advent of the direct antiviral agents (DAAs) there was a remarkable revolution on the treatment of hepatitis C, leading to very high rates of cure, varying from 85–100% [3] worldwide and 85–97% in Brazilian patients [4]. Even those patients not achieving sustained virological response (SVR) can be further submitted to retreatment also with very high rates of viral eradication [5].

However, it is presumed that a large amount of HCV + patients have been lost to follow-up (LTFU) in tertiary reference centers before the emergence of interferon-free regimens,compromisingWorld Health Organization (WHO) goals to achieve HCVeliminationas a public health problem worldwide until 2030 [6]. Even before the disruption of the HCV cascade of care (CoC) consequent to the COVID-19 pandemic, [7,8] investigators from the Polaris Observatory have estimated a significant delay in national action plans for hepatitis C eliminationin Brazil as well as in many countries [9].

Therefore, many initiatives are being conducted to carry on HCV elimination, some of them based on the adoption of different micro-elimination strategies. One approach that has been conducted elsewhere, with different success rates, [10–12] was focused on relinking patients LTFU after the identification of HCV infection in HCV outpatient’s clinics or hemodialysis units. Many were lost at different stages of HCV CoC before achieving SVR in primary care or even in hepatology reference centers in the last 5 to 10 years.

The aim of this study was to identify and retrieve patients LTFU in large reference centers in Brazil in order to offer them HCV treatment with the current highly effective DAAs. We also aimed at identifying possible reasons why these patients had not been previously treated and/or achieved SVR.

## Patients and methods

From May 2021 to May 2022, medical records of three outpatient clinics in Brazil were retrospectively reviewed to identify all HCV + subjects. The instrument used to review the charts was a standardized questionnaire including these information: name of the site, date of birth, sex, date of the last visit, presence or absence of cirrhosis, presence or absence of hepatocellular carcinoma, treatment status (treated or not treated), type of treatment (IFN or DAA), response to treatment and associated conditions (HIV, dialysis, transplant). The diagnosis of cirrhosis was made by liver biopsy or elastography higher than 12.5 kPa or clinical evidence of cirrhosis (esophageal varices or image showing a cirrhotic liver).

The participating centers were large reference facilities from the Federal University of São Paulo (São Paulo, SP), Bonsucesso Federal Hospital (Rio de Janeiro, RJ) and the Specialized Center for HCV Care of Salvador (Salvador, BA). All have been active for more than 10 years, providing healthcare free of charge through the Unified Health System(SUS) of Brazil. Healthcare personnel were trained to perform medical record review in a standardized way under the supervision of one attending physician in charge in each HCV outpatient clinics (local investigators).

Whenever an HCV-RNA + patient was encountered, lack of treatment or reasons for non-achievement of SVR were reported in the questionnaire: lost to follow-up, have already been treated with no response, not willing to be treated or not be eligible for treatment. Patients with diagnosis of cirrhosis were compared to non-cirrhotics regarding the frequency of being LTFU and the rate of non-response to treatment. All patients who were not treated or cured were contacted fora medical appointment at the discretion of the local investigator. Patients with severe comorbidities and short life expectancy were excluded from the study. All patients eligible to treatment were initially contacted by phone calls (up to three per patient) by the local investigator. In case of contact failure, new attempts were made by SMS or WhatsApp and then by regular mail using the residence address informed upon patient’s registry. All contacted patients were invited to schedule an appointmentin their clinic to be relinked to the HCV CoC. Treatments available at the time of the study in Brazil offered free of charge by SUS were sofosbuvir/ledipasvir for patients with genotype 1 or 4 and sofosbuvir/velpatasvir for patients with genotype 2 or 3.

All patients provided informed consent to participate in the study. The study was approved by the Ethical Committee from Federal University of Sao Paulo (number5.168.251).

### Statistical analysis

Continuous variables were expressed as mean and standard deviation, or as median and interquartile range (IQR) if skewed distribution. Categorical variables were expressed as absolute number and percentage.Categorical variables were compared between groups using Chi-squared test. The IBM SPSS Statistics24 software (IBM Corporation, NY, USA) was used for statistical analysis. All tests were two-tailed and a P value < 0.05 was considered significant.

## Results

Ten thousand two hundredeighty-nine HCV + patients (50% males, mean age: 52 ± 11 years) were identified, 4929 (54%) in SP, 3560 (36%) in RJ and 1800 (10%) in BA. Most patients (94%) had more than 40 years of age. Advanced fibrosis or cirrhosis and HCC were recorded in 2639 (26%) and 196 (2%) of those patients, respectively.

**Fig. 1:**
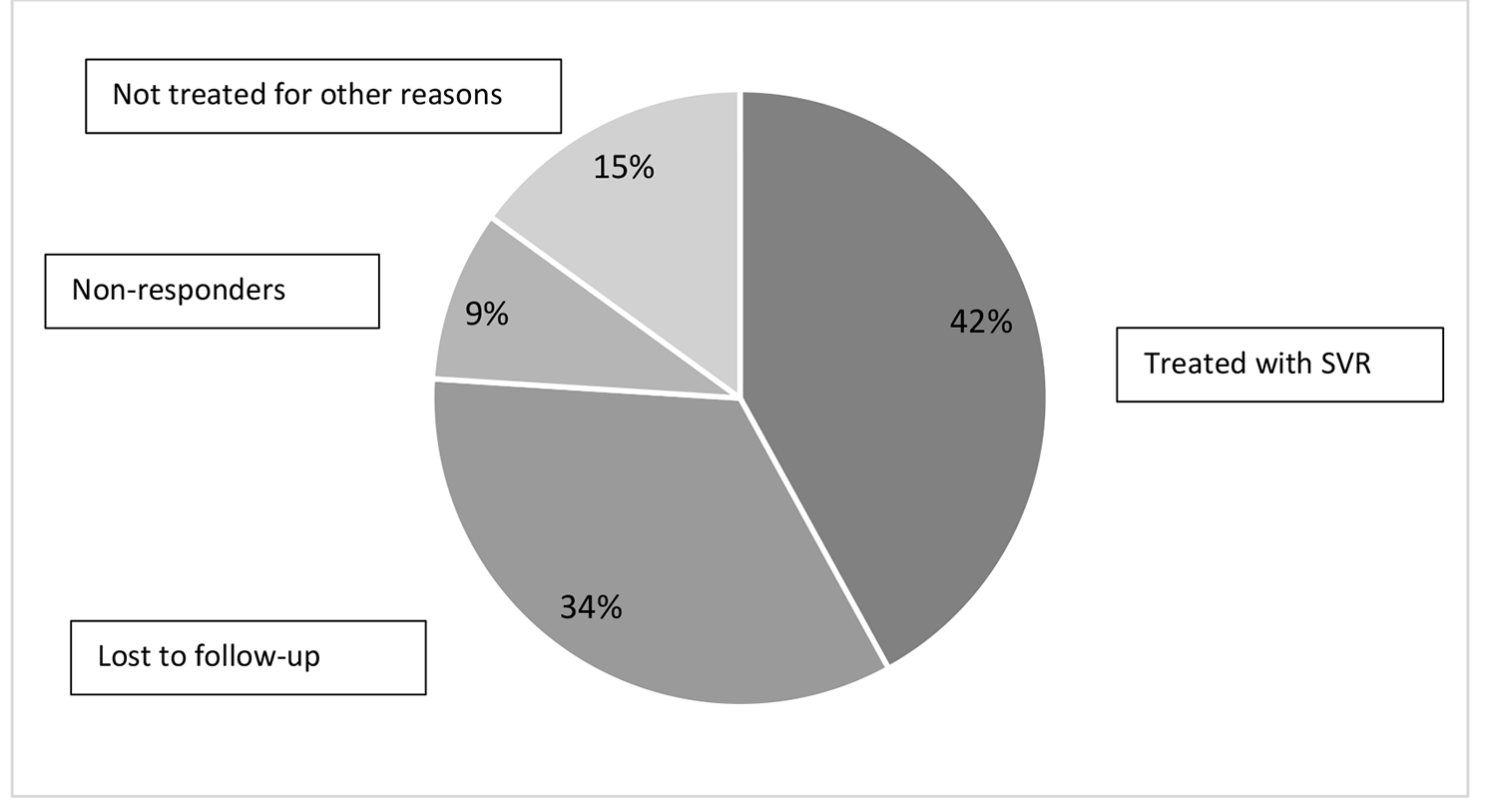
Characteristics of 10,289 anti−HCV positive patients retrospectively found in three reference centers in Brazil and the status of hepatitis C treatment

From these 10,289 patients evaluated only 4,293 (42%) had been previously treated and achieved SVR, 3,498 (34%) were LTFU and 1,543 (15%) were not treated due to other reasons. No response to interferon (IFN) based therapy or DAAs was observed in 926 patients (9%)(Fig. 1). Regarding the type of treatment, 814 were non-responders to INF and 112 to DAAs.In the group of cirrhotic patients (n = 2,639), 48% had been successfully treated, 24% were LTFU and 14% were non-responders. Comparing the reasons of not achieving SVR between cirrhotic patients and non-cirrhotics there was a significant difference in the frequency of no response to previous treatment (7% in non-cirrhoticsvs. 14% in cirrhotics, p < 0.05). The frequency of LTFU was also different between groups (41% in non-cirrhotics vs. 24% in cirrhotics, p < 0.05).

At local investigators discretion, 3,559 subjects were considered eligible to be retrieved. Overall, 1818 (51%) subjects could be contacted by phone calls (rate of callback of 18%), SMS or WhatsApp messages (13% callback) and regular mail (7% callback). (Table 1).

**Table 1:** – Patient retrieval strategies for patients with no registry of SVR and the success of each strategy

	Total	Phone calls	SMS	Whatsapp	Mail
Number of contact attempts	21,013	18,949	1368	361	335
Effective contact attempts	3559	3470	26	39	24
Callback rates (%)	17%	18%	2%	11%	7%
Contact feedback	1818	1758	26	17	24
Dead or transplanted	234	228	4	2	0
Accepted appointment	940	890	19	7	24
Treated elsewhere	454	450	3	1	0
Undergoing evaluation or treatment	106	106	0	0	0
Not willing to be contacted	84	84	0	7	0

**Fig. 2:**
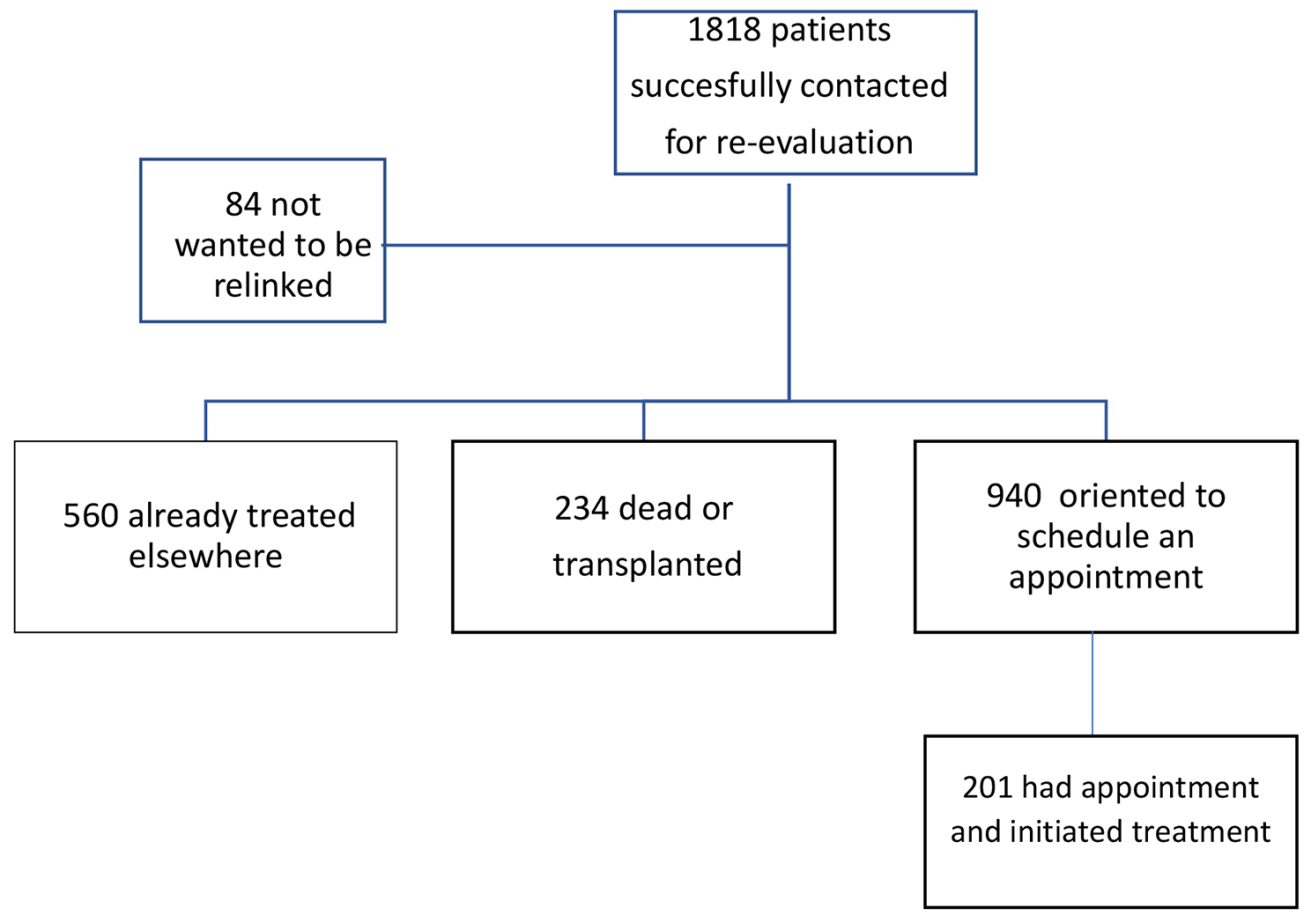
Retrieval flow-chart of 1818 HCV-patients successfully contacted for re-evaluation in three reference centers in Brazil

From these 1,818 contacted subjects, 560 patients have already been treated or were on treatment, 234 were reported to be dead or transplanted and 84 refused to be retrieved. From the remaining 940 patients, 739 patients were successfully contacted and oriented to schedule an appointment but didn’t do it in the six-month interval until the end of the study. Finally, 201 had made an appointment and initiated antiviral treatment (Fig. 2).

## Discussion

Our results showed that only 42% of the patients with chronic hepatitis C attending three reference centers for HCV treatment in Brazil have achieved SVR. Most of the remaining patients were untreated due to LTFU, comorbidity, unknown reasons, or the presence of mild disease. Furthermore 9% had no SVR due to treatment failure with IFN-based therapy or DAAs.

Those findings could be attributed to several factors, particularly the evolving policies for HCV treatment adopted by the Brazilian government over the last 5 to 10 years and the several barriers in access to healthcare, diagnosis and treatment despite public coverage of all aspects of HCV CoC though the Unified Health System (SUS). Thehighly effective DAAs approved for HCV treatment in Brazil were initially offered free of charge only for subjects with advanced fibrosis and cirrhosis. Universal treatment of all patients with active HCV infection and HCV CoC management and treatment simplification were implemented in Brazil only in 2019 and 2021, respectively, due to financial constraints [13]. Unavailability of DAAs for most of the patients with chronic hepatitis C for this long period of time could in part explain why so many patients were left untreated or LTFU. Lack of awareness of the consequences of the disease and fear of the common side effects of the IFN therapy [14] may also have impacted their retention in care. Finally, several steps and barriers during diagnosis and treatment of this disease in Brazil, including lack of access to genotyping and specialty clinics for HCV treatment, may have forced many patients to abandon healthcare.

Considering these mentioned reasons, it became clear that simplification of HCV treatment is crucial to retain patients in the health system until the achievement of SVR. Other studies have demonstrated that few patients initially diagnosed with hepatitis C achieved SVR due to the many steps in the CoC [15,16].

In the present study, 3,559 patients with chronic hepatitis C were eligible to be retrieved. Unfortunately,only half of them could be contacted particularly due to frequent changes in patient’s mobile phone numbers over time. Of 1,818 effectively contacted patients 794 (44%) were either treated elsewhere, dead or transplanted. After the attempts to schedule an appointment only 201 out of those 1,818 contacted subjects were up to now relinked to care.

Several different strategies were adopted worldwide for relinking patients with chronic hepatitis C who were either untreated, LTFU or failed HCV treatment with variable results regarding relinking to care, treatment and HCV SVR rates. Two previous reports from the Netherlands [11-12], and one from Taiwan [10] identified hundreds of patients LTFU reviewing data either from laboratory and/or medical files. Fewer patients were retrieved in the West, when compared to the East, but most of the patients relinked underwent treatment. In a study using a similar but extended approach involving 45 different healthcare facilities in the Netherlands [17], the authors identified 1,537 (8%) subjects out of 20,183 ever-diagnosed patients with hepatitis C that were LTFU and eligible for retrieval. Contact was established with 888/1,537 patients and 251 were referred for re-evaluation. Finally, 123 started antiviral therapy. The authors suggested that micro-elimination through retrieval could turn out to be an important strategy for worldwide HCV elimination. Similarly, a study from Spain [18] identified 166 HCV + patients eligible to be retrieved. Most of them were relinked despite the COVID-19 pandemic’s disruption of healthcare. Using Markov modeling, the authors additionally reported their micro-elimination strategy was cost-effective, that despite the low rates of patient retrieval. Another large study from Latin America was recently published involving 45 HCV reference centers throughout the continent including five different tertiary care centers from Brazil [19]. When compared to the present study, the authors reported similar percentages of patients LTFU as well as of HCV + subjects amenable to be retrieved. Results regarding relinking and treatment of those subjects are still pending.

This study has some limitations. The data were retrospectively collected from charts in three different centers, but a standardized questionnaire for collecting the data was used to minimize possible differences among the centers. Another limitation is the fact that the study was done in large reference centers and the results cannot be extrapolated to primary or secondary care, where the rates of LTFU patients are probably even higher.

In summary, our findings reinforce that despite the low rate of callback of different strategies to find previously diagnosed but untreated patients, micro-elimination through retrieval of patients LTFU is feasible and may contribute to HCV elimination particularly in countries where a large number of HCV + subjects were left untreated.

## Data Availability

The datasets used or analysed during the current study are available from the corresponding author on reasonable request.
